# Dapagliflozin as an adjunct to insulin improves glycemic control, weight, and lipid profile with a superior safety profile in type 2 diabetes: a randomized trial

**DOI:** 10.1016/j.clinsp.2026.101116

**Published:** 2026-09-06

**Authors:** Lili Sun, Guiling Liu, Baozhong Liang, Houmei You

**Affiliations:** a Taihe Hospital of Chinese Medicine Affiliated to Anhui University of Chinese Medicine, Fuyang, China; b Lexibao Health Management Co., Ltd., Beijing, China; c School of Chinese Medicine, Beijing University of Chinese Medicine, Beijing, China; d Beijing University of Chemical Technology, Beijing, China

**Keywords:** Type 2 diabetes, Dapagliflozin, Insulin therapy, Metabolic control, Randomized controlled trial

## Abstract

**Objective:**

To investigate the efficacy and safety of dapagliflozin-insulin combination in patients with Type 2 Diabetes Mellitus (T2DM).

**Methods:**

In this 3-month, randomized controlled trial, 86 T2DM patients were allocated to either insulin monotherapy (Group A) or dapagliflozin-insulin combination (Group B). Primary endpoints included glycemic control (Fasting Blood Glucose [FBG], 2-hour Postprandial Blood Glucose [2hPBG], Hemoglobin A1c [HbA1c]), lipid profile (Total Cholesterol [TC], Triglycerides [TG], Low-Density Lipoprotein Cholesterol [LDL-C], High-Density Lipoprotein Cholesterol [HDL-C]), anthropometrics (Body Mass Index [BMI], Body Weight [BW]), blood pressure (Systolic Blood Pressure [SBP], Diastolic Blood Pressure [DBP]), and adverse events.

**Results:**

After treatment, the combination therapy (Group B) demonstrated significantly greater improvements than insulin alone (Group A). Reductions in Group B versus Group A were: HbA1c (24.9%vs. 10.3%), FBG (23.6%vs. 5.6%), 2hPBG (25.1%vs. 13.7%), TC (35.1%vs. 16.9%), TG (15.9%vs. 11.0%), LDL-C (15.7%vs. 6.5%), SBP (7.3%vs. 3.2%), DBP (12.6%vs. 7.8%), BMI (9.8%vs. 4.3%), and BW (5.4%vs. 3.3%); while HDL-C increased more in Group B (16.3%vs. 9.9%). Group B demonstrated superior benefits in glycemic control, lipid profile, anthropometrics parameters and blood pressure reductions. All post-treatment between-group comparisons were statistically significant (p < 0.05). Critically, the incidence of adverse events was 71.4% lower in Group B, a statistically significant difference (p = 0.007).

**Conclusions:**

Dapagliflozin as an adjunct to insulin provides superior multi-faceted metabolic benefits encompassing glycemic control, weight reduction, lipid improvement, and blood pressure lowering, coupled with a more favorable safety profile, supporting its inclusion in standard insulin regimens for T2DM.

## Highlights

• Dapagliflozin added to insulin provided superior HbA1c reduction (–24.9%) vs. insulin alone (–10.3%).
• The combination therapy significantly improved lipid profile and reduced body weight and blood pressure.
• Adverse events were 71.4% lower with dapagliflozin-insulin, underscoring an enhanced safety profile.
• No cases of ketoacidosis occurred, supported by structured preventive measures.

## Introduction

Diabetes mellitus is a globally widespread chronic multi-causal metabolic disorder, primarily categorized as type 1 and type 2 diabetes mellitus. According to the International Diabetes Federation (IDF), approximately 589 million adults worldwide had diabetes in 2024, with this number projected to reach 853 million by 2050.^1^ China has the world’s largest diabetic population. The adult prevalence is as high as 12.8%, predominantly Type 2 Diabetes Mellitus (T2DM). Diabetes mellitus predisposes individuals to metabolic abnormalities, causing vascular, microvascular, neurological and other lesions thereby increasing the risk of cardiovascular and cerebrovascular diseases, even accompanied by chronic complications such as kidney disease, retinopathy, neuropathy, lower extremity vasculopathy, which seriously threaten the life safety of patients.^2^^,^^3^ Blood glucose control and improvement of insulin resistance are primary clinical treatments of T2DM. While many kinds of hypoglycemic drugs exist only ∼50% prove effective due to individual variability. At present, the treatment of T2DM heavily depends on insulin regulation, which directly stimulates pancreatic β-cells to secrete insulin or enhances insulin action to reduce blood glucose.^4^ Despite effectively controlling blood glucose, insulin has limited impact on blood pressure and lipid levels. It also causes side effects including nausea, vomiting, diarrhea, weight gain, and hypoglycemia.^5^^,^^6^ Therefore, it is urgent to find a combination of drugs to control blood glucose while minimizing side effects.

Sodium-Glucose Cotransporter 2 inhibitors (SGLT2i) play a key role in renal glucose reabsorption and have been used as a novel strategy for the treatment of T2DM.^6^ Results from the CVD-REAL and the EMPUSLE trials reported by the American College of Cardiology (ACC) and the results of a large randomized controlled study in 2022 showed that the treatment of T2DM patients with SGLT2i such as dapagliflozin significantly reduced the risk of hospitalization and death, and reduces the disturbance of pancreatic β-cell function and insulin resistance, leading to the effective treatment of T2DM patients.^7^^,^^8^ Dapagliflozin, as the first approved SGLT2i in China, inhibits the reabsorption of glucose in renal tubules and facilitates glucose excretion through urine, thereby reducing blood glucose levels continuously, stably and effectively over a long period of time in a non-insulin-dependent manner. Dapagliflozin also has a diuretic effect, reducing fluid retention, and in turn positively affecting blood pressure.^9^^,^^10^ In addition, dapagliflozin also offers cardiovascular protection by lowering blood pressure, blood lipids and serum uric acid levels, increasing cardiac ejection fraction, and aiding weight reduction, thereby improving metabolic disorders and reducing the occurrence of cardiovascular diseases.^11^, ^12^, ^13^, ^14^, ^15^, ^16^ According to the 2023 ADA/EASD consensus, SGLT2i are recommended as first-line therapy for T2DM, particularly advocating for early initiation of combination regimens when monotherapy proves inadequate.^17^ Although dapagliflozin, as a common clinical hypoglycemic drug, is effective in reducing blood glucose levels in patients, long-term clinical therapeutic practice has observed that the efficacy produced by the application of dapagliflozin alone has certain limitations, and there are few reports on clinical studies combined with dapagliflozin. Therefore, there is an urgent need to explore effective drug combination strategies to enhance the clinical treatment effect.

The combination of dapagliflozin and insulin is an important treatment strategy. In this study, we utilized dapagliflozin, which reduces patients’ blood glucose levels via a non-insulin-dependent mechanism, in combination with insulin to treat patients with T2DM. Both synergistic treatments demonstrated remarkable clinical efficacy in T2DM patients, particularly regarding their effects on blood pressure, blood lipids and weight management.

## Materials and methods

### Data source and study population

Ninety patients with T2DM admitted to our hospital from January 2021 to January 2024 were enrolled. Based on the randomized numerical table method, patients were randomly assigned to Group A (Insulin only) or observation Group B (Insulin + Dapagliflozin) using a computer-generated random number sequence with allocation concealment. The sample size was determined a priori by power analysis (G*Power 3.1): assuming an effect size of 0.8 for glycemic control improvement, α = 0.05, β = 0.045, a sample size of 86 patients would be required, with 43 cases in each group. To account for potential attrition, a total of ninety patients were enrolled in the current study. There was no significant difference between the both groups in general data (p > 0.05). The study was approved by the hospital ethics committee (2023LCTH05). The study was conducted following the STROBE statement and in accordance with the Declaration of Helsinki.^18^

### Inclusion and exclusion criteria

Inclusion criteria: 1) Meeting diagnostic criteria per *Chinese Guideline for Type 2 Diabetes* (2017)^19^; 2) Ejection Fraction (EF) > 50%; 3) No mental disorders; 4) Patient informed consent.

Exclusion criteria: 1) Presence of respiratory comorbidities associated with dyspnea or chest discomfort (e.g., chronic bronchitis, emphysema, pneumothorax, asthma); 2) Exclude heart disease caused by cardiomyopathy, valvular disease, right heart disease, arrhythmia and thyroid dysfunction; 3) Patients with severe abnormalities of liver and kidney function, blood system and other organs; 4) Urinary tract infection.

### Intervention protocols

Group A (Insulin only): Received insulin (Ganshulin 30R/40R, Tonghua Dongbao Pharmaceutical Co.) by subcutaneous injection. Initial dose: 0.2 U/kg twice daily, adjusted the dosage according to the patient’s blood glucose level to ensure that the blood glucose was controlled within the normal range.

Group B (Insulin + Dapagliflozin): Received identical insulin regimen as Group A ‌plus oral dapagliflozin (AstraZeneca, 10 mg/tablet), 10 mg once daily.

Both groups received treatments during hospitalization (7‒14 days) and continued for 3-months post-discharge via outpatient follow-up.

### Statistical methods

1) Blood glucose index: The levels of Fasting Blood Glucose (FBG) and 2-hours Postprandial Blood Glucose (2hPBG) were detected by blood glucose analyzer before and after treatment in both groups. 4 mL morning venous blood of the patients was taken, followed by centrifugation at 3000 rpm for 10 min to separate serum components. The level of Hemoglobin A1c (HbA1c) was detected by high performance liquid chromatography.^20^
2) Lipid index: Triglyceride (TG), total Cholesterol (TC), Low-Density Lipoprotein Cholesterol (LDL-C), and High-Density Lipoprotein Cholesterol (HDL-C) were measured before and after the treatment in both groups employing a fully automatic biochemical analyzer (Beckman DXC800).^21^
3) Blood pressure index: Systolic Blood Pressure (SBP) and Diastolic Blood Pressure (DBP) were measured by sphygmomanometer.
4) Body Weight (BW) and Body Mass Index (BMI): The BW of patients for the both groups was measured using an electronic weight scale, and BMI was calculated by BMI = weight(kg)/height(m)^2^.
5) Adverse reactions: Observe the occurrence of adverse reactions, mainly including urinary tract infection, hypoglycemia, skin rash, diarrhea and so on.

### Data analysis

Analysts were blinded to group allocation during outcome assessment. ‌SPSS20.0 statistical software was used for analysis. The measurement data included blood glucose index, blood lipid index, blood pressure index, BW and BMI, which were expressed as mean ± standard deviation ($\bar{x}$±s) and compared via *t*-tests. Categorical data (adverse reaction rates) were compared using χ^2^-tests; p < 0.05 indicated that the difference was statistically significant.

## Results

### Comparison of blood glucose index in the both groups before and after treatment

Table 1 shows the changes in FBG, 2hPBG and HbA1c parameters after taking insulin only (Group A) and taking insulin + dapagliflozin (Group B). It can be observed that Group A exhibited reductions of 5.6% in FBG, 13.7% in 2hPBG, and 10.3% in HbA1c. Similarly, Group B demonstrated reductions of 23.6% in FBG, 25.1% in 2hPBG, and 24.9% in HbA1c.‌ FBG, 2hPBG and HbA1c reductions were significantly greater in Group B than in Group A, indicating superior blood glucose index control. There was no significant difference in blood glucose index between the both groups before treatment (p > 0.05). The blood glucose index of the both groups after treatment was lower than those before treatment, while the blood glucose index of the B was lower than those of the A. The difference was statistically significant (p < 0.05), indicating that the combination of dapagliflozin and insulin could more effectively reduce the blood glucose level. The results are shown in Table 1 and expressed as ($\bar{x}$±s).

**Table 1:** Comparison of blood glucose index in the both groups before and after treatment.

	Treatment	A	B	*t*	p
**FBG (mmoL/L)**	Before	9.26±2.06	9.31±2.10	0.111	0.912
	After	8.74±1.09^a^	7.11±1.08^a^	6.966	<0.001
**2hPBG (mmoL/L)**	Before	10.62±1.32	10.70±1.27	0.286	0.775
	After	9.17±1.12^a^	8.01±1.04^a^	4.976	<0.001
**HbA1c (%)**	Before	8.45±1.15	8.27±1.07	0.751	0.454
	After	7.58±1.08^a^	6.21±1.01^a^	6.075	<0.001

Note: A, Insulin only; B, Insulin + dapagliflozin; FBG, Fasting Blood Glucose; 2hPBG, 2-hours Postprandial Blood Glucose; HbA1c, Hemoglobin A1c. Paired *t*-tests for within-group comparisons, independent *t*-tests for between-group comparisons.

a p < 0.05 is considered significantly different for pre-post treatment.

### Comparison of blood lipid index in the both groups before and after treatment

Table 2 shows the changes in TC, TG, LDL-C and HDL-C parameters after taking insulin only (Group A) and taking insulin + dapagliflozin (Group B). Group A exhibited reductions of 16.9% in TC, 11.0% in TG, and 6.5% in LDL-C, along with a 9.9% increase in HDL-C. Similarly, Group B showed greater reductions (35.1% in TC, 15.9% in TG, 15.7% in LDL-C) and a higher HDL-C increase (16.3%). The reductions in TC, TG, and LDL-C, along with the increase in HDL-C, were significantly greater in Group B than in Group A, suggesting better lipid profile management which may contribute to improved glycemic control. There was no significant difference in blood lipid index between the both groups before treatment (p > 0.05). After treatment, the levels of TC, TG and LDL-C in the both groups were lower than those before treatment, and HDL-C was higher than that before treatment. The levels of TC, TG and LDL-C in the B were lower than those in the A, and HDL-C level was higher than that in the A. The difference was statistically significant (p < 0.05), indicating that the combination of dapagliflozin and insulin had a positive effect on weight management. The results are shown in Table 2 and expressed as ($\bar{x}$±s) (mmoL/L).

**Table 2:** Comparison of blood lipid index in the both groups before and after treatment.

	Treatment	A	B	*t*	p
**TC**	Before	2.54±0.21	2.48±0.23	1.263	0.21
	After	2.11±0.17^a^	1.61±0.11^a^	16.192	<0.001
**TG**	Before	4.81±0.41	4.77±0.44	0.436	0.664
	After	4.28±0.37^a^	4.01±0.31^a^	3.667	0.001
**LDL-C**	Before	3.54±0.31	3.57±0.30	0.456	0.649
	After	3.31±0.25^a^	3.01±0.21^a^	6.025	<0.001
**HDL-C**	Before	1.01±0.13	1.04±0.11	1.155	0.251
	After	1.11±0.17^a^	1.21±0.18^a^	2.648	0.009

Note: A, Insulin only; B, Insulin + dapagliflozin; TC, Total Cholesterol; TG, Triglyceride; LDL-C, Low-Density Lipoprotein Cholesterol; HDL-C, High-Density Lipoprotein Cholesterol. Paired *t*-tests for within-group comparisons, independent *t*-tests for between-group comparisons.

a p < 0.05 is considered significantly different for pre-post treatment.

### Comparison of blood pressure index, BW and BMI in the both groups before and after treatment

Table 3 compares parameter changes between insulin monotherapy (Group A) and insulin-dapagliflozin combination therapy (Group B). Group A showed reductions of 3.2% in SBP, 7.8% in DBP, 4.3% in BMI and 3.3% in BW, while Group B demonstrated significantly greater reductions (7.3% in SBP, 12.6% in DBP, 9.8% in BMI and 5.4% in BW). These metabolic improvements in Group B suggest potential synergistic effects on both hemodynamic parameters and body composition. There was no significant difference in blood pressure index, BW and BMI between the both groups before treatment (p > 0.05). The blood pressure index, BW and BMI of the both groups after treatment were lower than those before treatment, and the blood pressure index, BW and BMI of the B were lower than those of the A, the differences were statistically significant (p < 0.05). The systolic blood pressure, diastolic blood pressure, BW and BMI of the patients in the B decreased significantly, indicating that the synergistic effect of dapagliflozin and insulin can reduce the blood glucose level while reducing the blood pressure and the BW of patients. The results are shown in Table 3 and expressed as ($\bar{x}$±s).

**Table 3:** Comparison of blood pressure index, BW and BMI in the both groups before and after treatment.

	Treatment	A	B	*t*	p
**SBP (mmHg)**	Before	124.71±6.20	124.46±5.72	0.194	0.846
	After	120.71±6.46^a^	115.41±5.65^a^	4.049	<0.001
**DBP (mmHg)**	Before	80.27±5.46	80.57±5.41	0.255	0.798
	After	74.02±5.40^a^	70.41±5.63^a^	3.034	0.003
**BMI (kg·m^−2^)**	Before	28.06±2.02	27.72±0.62	1.055	0.294
	After	26.84±2.01^a^	25.01±0.62^a^	5.704	<0.001
**BW (kg)**	Before	83.78±2.40	83.36±2.78	0.749	0.455
	After	81.01±2.34^a^	78.87±2.85^a^	3.805	<0.001

Note: A, Insulin only; B, Insulin + dapagliflozin; SBP, Systolic Blood Pressure; DBP, Diastolic Blood Pressure; BMI, Body Mass Index; BW, Body Weight. Paired *t*-tests for within-group comparisons, independent *t*-tests for between-group comparisons.

a p < 0.05 is considered significantly different for pre-post treatment.

### Comparison of adverse reaction incidence between the both groups

Safety profiles demonstrated significant divergence between Group A (insulin only) and Group B (insulin + dapagliflozin), each comprising 43 participants. The overall adverse event incidence‌ was markedly higher in Group A (32.56%, 14/43) compared to Group B (9.30%, 4/43). Dapagliflozin-insulin combination therapy can effectively lower adverse events (71.4%‌, p = 0.007), with statistical significance confirmed by Chi-Square test (χ^2^ = 7.298, p = 0.007), indicating that dapagliflozin combined with insulin in the treatment of patients with T2DM can effectively reduce side effects such as hypoglycemia caused by insulin alone, demonstrating the great potential of dapagliflozin combined with insulin in the treatment of T2DM. Notably, no cases of Diabetic Ketoacidosis (DKA), including euglycemic DKA, were observed in either group during the study period. To proactively mitigate the recognized risk of euglycemic DKA associated with SGLT2i,^17^ we implemented the following preventive measures throughout the study for all participants in Group B: 1) Patient education regarding DKA symptoms and the importance of seeking immediate medical attention; 2) Guidance for ketone monitoring (blood or urine) during periods of acute illness, sustained hyperglycemia, or symptoms suggestive of DKA; 3) Temporary suspension of dapagliflozin during acute illness or planned surgical procedures per protocol; 4) Individualized insulin titration with close monitoring to avoid significant insulin dose reduction; and 5) Emphasis on maintaining adequate hydration. The results are shown in Table 4 and expressed as n (%).

**Table 4:** Comparison of adverse reaction incidence between the both groups.

	A	B	χ^2^	p
**Number**	43	43	‒	‒
**Urinary tract infection**	2	0	‒	‒
**Hypoglycemia**	5	2	‒	‒
**Skin rash**	3	1	‒	‒
**Diarrhea**	4	1	‒	‒
**Total incidence**	14 (32.56)	4 (9.30)	7.298	0.007

Note: A, Insulin only; B, Insulin + dapagliflozin. The χ^2^ test was used to analyze differences between groups. χ^2^ test with Yates’ correction applied for overall comparison. ^a^ p < 0.05 is considered significantly different for pre-post treatment.

### Comparative analysis

As summarized in Table 5, our findings regarding significant HbA1c reduction and weight benefit with dapagliflozin added to insulin are consistent with trials such as Araki E,^22^ Chen JF^9^ and Hamza A.^23^ Notably, the markedly lower overall adverse event incidence (9.30%), observed in our combination group compared to both our insulin-only group and the rates reported in Araki E and Chen JF (Table 5), further strengthens the safety advantage of this regimen in our patients. This difference may be attributed to stricter preventive measures.

**Table 5:** Comparative analysis of key findings with selected prior studies.

Study source	This research	Araki E^22^	Chen JF^9^	Hamza A^23^
Study duration (weeks)	12	52	24	12
Study arms	Insulin / Insulin - Dapagliflozin	Dapagliflozin / Placebo-Dapagliflozin	Dapagliflozin	Dapagliflozinn in IRS / RID
Randomized participants, N	86	182	1960	180
Baseline insulin dose	0.2 U/kg	≥0.2 U/kg	/	/
Baseline dapagliflozin dose	10 mg	5∼10 mg	5∼10 mg	10 mg
Hba1c reduction (%)	10.3% / 24.9%	10.28% / 11.53%	11.36%	15.38% / 12.05%
BW reduction (kg)	2.77 kg / 4.49 kg	1.5 kg / 1.37 kg	1.61 kg	2.2 kg / 3.5 kg
Overall AE rate (%)	32.56% / 9.3%	82.9% / 71.7%	20.41%	5.5% / 5.5%

Note: IRS, Insulin Resistance Syndrome; RID, Relative Insulin Deficiency; HbA1c, Hemoglobin A1c; BW, Body Weight; Overall AE rate, Overall Adverse Event rate.

This comparative analysis (Table 5) underscores that our study not only corroborates the established efficacy of dapagliflozin-insulin combination but also provides compelling evidence for its superior tolerability profile in terms of reduced overall adverse events within the context of specific population.

## Discussion

T2DM represents a prevalent endocrine and metabolic disorder characterized by insulin resistance, inadequate insulin secretion, and progressive β-cell dysfunction, leading to dysregulation of macronutrient metabolism and elevated risks of cardiovascular and renal complications.^24^^,^^25^ Genetic predispositions, lifestyle factors, and obesity contribute significantly to this pathogenesis, with obesity exacerbating insulin resistance and necessitating higher insulin doses that further promote weight gain, dyslipidemia, and hypoglycemia. Current therapeutic strategies prioritize glycemic stabilization, symptom alleviation, and cardiovascular risk mitigation, where dapagliflozin ‒ a non-insulin-dependent SGLT2 inhibitor ‒ emerges as a key agent by lowering blood glucose, reducing Body Weight (BW), and conferring cardiorenal protection.^3^^,^^15^^,^^20^^,^^21^^,^^24^^,^^26^ Evidence increasingly supports that combining dapagliflozin with insulin enhances long-term glycemic control and multidimensional management of T2DM complications, as demonstrated in trials like EMPA-REG OUTCOME, DECLARE-TIMI 58 and DAPA-HF.^3^^,^^15^^,^^20^^,^^21^^,^^24^^,^^26^, ^27^, ^28^, ^29^

Glycemic stability is paramount for preventing diabetes complications. In this study, post-treatment blood glucose levels in Group B were significantly reduced compared to Group A (p < 0.05), attributable to the synergistic mechanisms of dapagliflozin-insulin therapy. Dapagliflozin inhibits SGLT2-mediated glucose reabsorption in renal proximal tubules, promoting urinary glucose excretion and reducing hyperglycemia. Concurrently, insulin facilitates glucose utilization via GLUT4 translocation in target tissues, compensating for β-cell failure.^25^ This combination amplifies endogenous insulin effects, improving insulin sensitivity and resistance, as corroborated by EMPA-REG OUTCOME and DECLARE-TIMI 58, where SGLT2i reduced HbA1c by 0.5%–0.8% versus placebo.^30^, ^31^, ^32^, ^33^

Dyslipidemia, a critical cardiovascular risk factor, showed marked improvement in Group B, with greater reductions in TC, TG, and LDL-C, alongside increased HDL-C (p < 0.05). This reflects dual pathways: dapagliflozin ameliorates insulin resistance-induced lipid metabolism disorders by suppressing lipolysis and free fatty acid release, while insulin promotes fatty acid synthesis and storage, attenuating dyslipidemia.^34^ Notably, DAPA-HF, EMPA-REG OUTCOME and DECLARE-TIMI 58 documented TC, TG and LDL-C reductions with SGLT2i, underscoring their role in mitigating atherosclerosis risk in T2DM patients with lipid abnormalities.

Weight and BMI reductions in Group B (p < 0.05) arise from dapagliflozin-induced caloric loss via glycosuria and insulin-mediated promotion of anabolic glucose storage, reducing ectopic fat deposition. This synergy is superior to insulin monotherapy, which often causes weight gain. Importantly, obesity exacerbates OSAHS-related cardiovascular risks, and our findings support combination therapy as a strategy to interrupt this cascade, aligning with outcomes from Asian Diabetes Outcomes Prevention Trial.^35^

The significantly reduced adverse events in Group B demonstrate the safety advantage of dapagliflozin-insulin therapy. Critically, no euglycemic DKA events occurred despite the known background risk (0.1%‒0.5%) associated with SGLT2i. This absence, achieved through proactive implementation of guideline-based preventive measures (e.g., ketone monitoring during acute illness, individualized insulin titration, and temporary SGLT2i suspension), supports the manageable nature of this risk within a structured protocol. These findings validate the combination as a viable strategy for optimizing T2DM safety outcomes. Continued adherence to mitigation strategies remains essential for long-term safety in insulin-treated patients.

Collectively, these findings validate the dapagliflozin-insulin regimen as a synergistic approach for comprehensive T2DM management, addressing glycemia, lipids, weight, and safety within the Cardiovascular-Renal-Metabolic (CKM) syndrome framework. Future studies should explore long-term outcomes using continuous glucose monitoring to refine therapeutic protocols.

## Conclusions

Dapagliflozin-insulin combination therapy significantly improves T2DM management by achieving superior glycemic control, optimizing lipid metabolism, reducing body weight/BMI and ‌Lowering adverse events. ‌This regimen warrants prioritization in clinical practice for T2DM management with obesity comorbidities.

## Data availability statement

The datasets generated and/or analyzed during the current study are available from the corresponding author upon reasonable request.

## Ethics statement

The present study and this post hoc analysis were approved by the Medical Ethics Committee of Taihe Hospital of Chinese Medicine Affiliated to Anhui University of Chinese Medicine (Approval No. 2023LCTH05). All procedures were performed in accordance with the ethical standards of the Institutional Review Board and The Declaration of Helsinki, and its later amendments or comparable ethical standards. Informed consent was obtained from all participants prior to their enrollment in the study.

## Declaration of generative AI and AI-assisted technologies in the writing process

The authors declare that CNKI Translation Assistant, DeepL, and Youdao Translation were used during the preparation of this manuscript exclusively to support language editing, grammar correction, and text organization. All content was carefully reviewed and validated by the authors, who assume full responsibility for the final version of the manuscript.

## Authors’ contributions

Lili Sun has made substantial contributions to the analysis of the data for the work, interpretation of the data for the work and critical revision of the manuscript for important intellectual content. Guiling Liu has made substantial contributions to the interpretation of the data for the work and critical revision of the manuscript for important intellectual content. Baozhong Liang has made substantial contributions to the conception of the work, interpretation of the data for the work and critical revision of the manuscript for important intellectual content. Houmei You has made substantial contributions to the conception of the work, interpretation of the data for the work and critical revision of the manuscript for important intellectual content.

## Funding

No funding was received for this research.

## Conflicts of interest

The authors declare no conflicts of interest.
